# Simple induction of pseudopregnancy by artificial stimulation using a sonic vibration in rats

**DOI:** 10.1038/s41598-020-59611-1

**Published:** 2020-02-17

**Authors:** Takehito Kaneko, Marina Endo, Shigemi Tsunoda, Yuki Nakagawa, Hisayuki Abe

**Affiliations:** 1 Division of Science and Engineering Graduate School of Arts and Science, Iwate University, Iwate, 020-8551 Japan; 2 Department of Chemistry and Biological Sciences, Faculty of Science and Engineering, Iwate University, Iwate, 020-8551 Japan; 3 Institute for Animal Reproduction, Ibaraki, 300-0134 Japan

**Keywords:** Infertility, Animal physiology

## Abstract

Embryo transfer has been used as one of the essential reproductive technologies for production of new strains and maintenance of genetic resources in animals. Mating with vasectomised male rats is a requirement for inducing pseudopregnancy in female rats selected for embryo transfer. Although this procedure has been used routinely, large breeding space and high expenditure are required to maintain a sufficient number of females and vasectomised males. This study was performed to induce pseudopregnancy in females by artificial stimulation using sonic vibration instead of vasectomised males. The females continued to be in the dioestrus stage for at least 14 days after artificial stimulation was performed. Of fresh 2-cell embryos that transferred into the oviducts of females after artificial stimulation, 56% was implanted and 50% was developed to offspring. Approximately 46% of the frozen 2-cell embryos were implanted and 24% developed into offspring. Furthermore, 66% of the fresh pronuclear embryos were implanted and 60% developed into offspring. This study successfully induced pseudopregnancy in rat females by artificial stimulation using a sonic vibration. This method, ‘Easy-ET’, was useful for efficient production and maintenance of rat strains.

## Introduction

Reproductive technologies have been developed for production of new strains and maintenance of genetic resources in animals^1^. Embryo transfer (ET) has been also established as one of the essential reproductive technologies in domestic and laboratory animals. ET was first studied in rabbits by Heape^2^. It was then studied in the oviducts or uterus of rabbit^3,4^, mouse^5,6^, and rat^7,8^ females. The ET protocol has remained unmodified since its first report. Presently, transfer of 2-cell embryos into oviducts is being routinely used as a stable standard technology for the efficient production of genetically engineered strains and removal of pathogens in laboratory animals.

Recently, many types of genetically modified rats have been produced to study human diseases^9,10^. Genome editing technology, clustered regularly interspaced short palindromic repeat (CRISPR)/CRISPR-associated (Cas) system, has further increased the frequency of production of genetically modified strains^11^. Various strains of the human disease model have been rapidly produced by simple endonuclease introduction technique into embryos using microinjection^12–14^ and the electroporation (TAKE - Technique for Animal Knockout system by Electroporation) method^15–17^. ET is required for the efficient production of new strains from these genome-edited embryos and the regeneration of valuable strains from frozen embryos that are used as genetic resources^18–20^.

In rats, 2-cell embryos are routinely transferred to the oviducts of females. The females require mating stimulation to maintain pregnancy. Pseudopregnancy is generally induced in proestrus female rats used for ET by mating with vasectomised male rats the day before ET is performed. In this procedure, maintenance of a sufficient number of healthy females and vasectomised males for induction of pseudopregnancy in proestrus females requires large breeding space and is expensive.

In this study, we successfully performed simple induction of pseudopregnancy by artificial stimulation using a sonic vibration instead of vasectomised males. The embryos transferred into the oviducts of these pseudopregnant females developed into healthy offspring.

## Results

Several pairs of matured male and a female at oestrus stage were monitored for their copulatory behaviour for 2 h in the dark at least 30 times using a video camera. Copulatory behaviour was repeated 5 to 7 times in 5 min intervals in dark. One copulatory behaviour, such as mount and intromission including ejaculation, lasted approximately 5 min. It was calculated that the time of stimulation by intromission during the one copulatory behaviour was about 30 s. Based on this result, the females were artificially stimulated by a sonic vibration for 30 s per stimulation 7 times in 5 min intervals.

The oestrus cycle of females showing soliciting behaviour at the proestrus stage after artificial stimulation by sonic vibration was continuously monitored (Table 1). In the control setup, females without artificial stimulation showed regular 4-days oestrus cycle. The oestrus cycle of each of the 10 proestrus females stimulated artificially was analysed by observation of vaginal smears. The females continued to be in the dioestrus stage for at least 14 days after artificial stimulation (Table 1). The results showed that pseudopregnancy was induced by artificial stimulation using sonic vibration.

**Table 1:** The daily change in the stages of oestrous cycle in female rats after artificial stimulation with a sonic vibration.

Rat no.	Day 0	Day 1	Day 2	Day 3	Day 4	Day 5	Day 6	Day 7	Day 8	Day 9	Day 10	Day 11	Day 12	Day 13	Day 14	Day 15	Day 16
Control	P	E	M	D	P	E	M	D	P	E	M	D	P	E	M	D	P
1	P	E	M	D	D	D	D	D	D	D	D	D	D	D	D	D	P
2	P	E	M	D	D	D	D	D	D	D	D	D	D	D	D	P	—
3	P	E	M	D	D	D	D	D	D	D	D	D	D	D	P	—	—
4	P	E	M	D	D	D	D	D	D	D	D	D	D	D	D	D	P
5	P	E	M	D	D	D	D	D	D	D	D	D	D	D	D	D	P
6	P	E	M	D	D	D	D	D	D	D	D	D	D	D	P	—	—
7	P	E	M	D	D	D	D	D	D	D	D	D	D	D	D	D	P
8	P	E	M	D	D	D	D	D	D	D	D	D	D	D	D	P	—
9	P	E	M	D	D	D	D	D	D	D	D	D	D	D	D	P	—
10	P	E	M	D	D	D	D	D	D	D	D	D	D	D	P	—	—

Stages of oestrus cycle: proestrus (P), oestrus (E), metestrus (M) or dioestrus (D). Artificial stimulation was carried out using females that showed soliciting behaviour at proestrus stage (Day 0). “–“ mean not tested

Table 2 shows the development of embryos to offspring after the transfer to oviducts of females who had undergone artificial stimulation-induced pseudopregnancy. A total of 56% of the fresh 2-cell embryos transferred into the oviducts of females after artificial stimulation were implanted and 50% developed into normal offspring. In the control setup, 73% of the fresh 2-cell embryos transferred into the oviducts of females mated with vasectomised males were implanted and 66% developed into normal offspring. No significant differences were shown in the development of fresh 2-cell embryos to offspring into the females after artificial stimulation compared to control females. In addition, 46% of the frozen 2-cell stage embryos transferred females after artificial stimulation were implanted and 24% developed into offspring. In the control using females mated with vasectomised males, 56% of the frozen 2-cell embryos were implanted and 32% developed into normal offspring. No significant differences were shown in the implantation and developmental rates to offspring of frozen 2-cell embryos into the females after artificial stimulation compared to control females. Furthermore, 66% of the fresh pronuclear embryos transferred were implanted and 60% developed into offspring. The development of fresh pronuclear embryos transferred to females after artificial stimulation to offspring showed no significant differences compared to that of fresh 2-cell embryos transferred into females induced pseudopregnant by artificial stimulation or vasectomised males.

**Table 2:** Development of embryos to offspring after transfer to oviducts of females with pseudopregnancy induced by artificial stimulation with a sonic vibration.

Pseudopregnant	Status of embryos	No. of females	No. of embryos transferred	No. (%) of embryos implanted	No. (%) of offspring
With vasectomised males	Fresh, 2-cell	4	80	58 (73)^a^	53 (66)^c^
	Frozen, 2-cell	3	66	37 (56)	21 (32)^d^
Artificial stimulation	Fresh, 2-cell	4	80	45 (56)^b^	40 (50)^c^
	Frozen, 2-cell	3	55	25 (46)^b^	13(24)^d^
	Fresh, pronuclear	4	80	53 (66)^a^	48 (60)^c^

Significant differences at P < 0.05: a vs. b; c vs. d.

## Discussion

In this study, we performed induction of pseudopregnancy by artificial stimulation using a sonic vibration instead of vasectomised males. The females continued to be in the dioestrus stage for at least 14 days after artificial stimulation. The pronuclear and 2-cell embryos transferred into the oviducts of females after artificial stimulation developed into normal offspring. Therefore, pseudopregnancy could be induced in rat females by artificial stimulation using a sonic vibration.

Pseudopregnancy is generally induced in proestrus females via mating with vasectomised males. The maintenance of a sufficient number of healthy females and vasectomised males requires a large space and is expensive. This maintenance of vasectomised males is not required for the induction of artificial pseudopregnancy using sonic vibration. DeFeo was successful the induction of pseudopregnancy in the rat by vaginal cervical vibration^21^. Our results were demonstrated the continuation of pregnancy and developmental ability of transferred embryos to offspring. Furthermore, the Iar:Wistar-Imamichi rats used in this study showed well-regulated 4-day oestrus cycle (Table 1). In our internal studies, we found that the 4-day oestrus cycle was well-regulated in 40 7–10 week old females (data not shown). This suggests that the animals used for the study can be economically bred without the need to maintain additional females to prepare proestrus females.

In this study, both pronuclear and 2-cell embryos were transferred into the oviducts of each female. Both developmental stage embryos have been used for ET because the production of genetically engineered strains using genome editing technology such as CRISPR/Cas system requires genome modification of the pronuclear embryos, and 2-cell embryos of many valuable strains have been frozen as genetic resources. Presently, the ET of 2-cell embryos into oviducts is being routinely used as a stable standard technology for the efficient production of laboratory animals. This study also showed stable results that a high proportion (50%) of 2-cell embryos transferred to females after artificial stimulation developed into normal offspring (Table 2). Normal offspring (60%) were also obtained from the pronuclear embryos transferred to females after artificial stimulation (Table 2). The efficient implantation of embryos and subsequent successful pregnancy requires optimal time of embryo transfer in females. It was known that the sensitive interaction between embryos and female conditions for implantation of embryos that called implantation window affected the success rate of implantation^22^. Furthermore, it is thought that the receptivity of the uterus for implantation is extremely limited in mice^23^. This study demonstrated that no significant differences were obtained in the development of pronuclear and 2-cell embryos transferred to females after artificial stimulation using sonic vibration. Genome editing rats were generally produced by transfer of embryos that developed to 2-cell stage in next days of genome editing to pronuclear stage embryos. Although further study is required to clarify the interaction between transferred embryos and female conditions after artificial stimulation for implantation, this result suggests that the transfer of pronuclear stage embryos made it possible to genome modification of pronuclear stage embryos and embryo transfer in one day.

Although the transfer of 2-cell embryos into oviducts of females is a standard technology, ET into the uterus is also used as a valuable technique for production of chimeric animals using embryonic stem cells and animals with difficulties in transferring embryos into oviducts. The uterine transfer could be possible because the oestrus cycle of females after artificial stimulation using sonic vibration is maintained at the dioestrus stage for at least 14 days (Table 1). Furthermore, this technique can also be applied in mice because the mechanism of pseudopregnancy in mice is similar to rats

In this study, we have successfully induced pseudopregnancy in female rats by artificial stimulation using a sonic vibration instead of vasectomised males. The embryos transferred into the oviducts of these pseudopregnant female rats developed into morphologically normal offspring. The procedure is economical as it does not involve large breeding spaces and expenses for inducing pseudopregnancy, and contributes to animal welfare and 3Rs by minimising the use of laboratory animals needed for performing it.

## Methods

### Animals

Iar:Wistar-Imamichi rats (Institute for Animal Reproduction, Ibaraki, Japan) were used for embryo collection and subsequent embryo transfer. The animals were maintained in plastic cages in an air-conditioned (temperature 23 °C ± 3 °C, humidity 50% ± 10%) and light-controlled room (illuminated from 07:00 to 19:00 h). All animal care and procedures performed in this study conformed to the Guidelines for Animal Experiments of Iwate University and Institute for Animal Reproduction and were approved by the Animal Research Committee of Iwate University and Institute for Animal Reproduction.

### Video monitoring of copulatory behaviour

Several pairs of matured male and a female at oestrus stage were monitored for their copulatory behaviour for 2 h in the dark using a video camera. The time taken for one copulatory behaviour, including mount, intromission, and ejaculation, was recorded. This analysis was continued at least 30 times using several pairs. The time for artificial stimulation by the sonic vibration and interval between stimulations were calculated based on these monitoring results.

### Embryo collection

Female rats aged 10–14 weeks were collected randomly, and induced to superovulate by injecting pregnant mare serum gonadotropin (PMSG; ASKA Animal Health Co., Ltd., Tokyo, Japan) at a dose of 300 IU/kg intraperitoneally followed by an intraperitoneal injection of human chorionic gonadotropin (hCG; ASKA Animal Health Co., Ltd.) at a dose of 300 IU/kg 48 h later. They were then mated overnight with mature males aged more than 10 weeks. Presence of vaginal plugs of females were confirmed that mating has occurred. The pronuclear embryos were collected by flushing the oviducts with modified Krebs-Ringer bicarbonate (mKRB) medium after 24 h of hCG injection^24,25^. Some embryos were then cultured to 2-cell stage in a fresh mKRB medium at 37 °C under 5% CO_2_ and 95% air concentrations.

### Embryo cryopreservation

The 2-cell embryos were frozen via the vitrification method^18^. The embryos were placed in a solution of 10% propylene glycol in PB1^26^ at room temperature for 10 min and then transferred in 5 μL aliquots into 1.2 mL serum tubes (Sumitomo Bakelite Co. Ltd, Tokyo, Japan). Tubes with embryos were cooled for 1 min at 0 °C, and 95 μL of a solution containing 10% propylene glycol, 30% ethylene glycol, 20% Percoll, and 0.3 M sucrose in PB1 was added into the tubes. After cooling at 0 °C for 1 min, tubes were directly plunged in liquid nitrogen.

For warming of embryos, the frozen tubes were held at room temperature for 30 s and 900 μL of a solution containing 0.3 M sucrose in PB1 warmed to 37 °C was added to the tubes. After mixing the solution by gentle pipetting, the embryos were collected and placed in PB1 medium. After warming, the survival rate and normality of embryos were estimated.

### Artificial pseudopregnant of females

**Figure 1:**
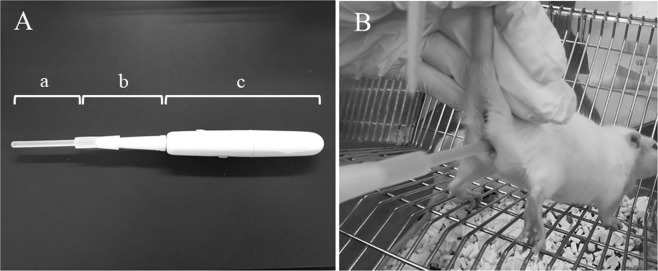
Sonic vibrator. (**A**) Fluororesin probe (a) and devise body (c) were connected by adaptor (b). Insertion of fluororesin probe in the vagina of female (**B**).

Induction of pseudopregnancy was carried out in proestrus females aged 10–14 weeks showed soliciting behaviour at 16:00–17:00 h. A self-made sonic vibrator (Fig. 1A) attached with a fluororesin probe (diameter: 5 mm, length: 7 cm) (Fig. 1A-a) was used for artificial stimulation. The probe of vibrator was inserted in the vagina of the female (Fig. 1B). and the tip was gently pressed to the cervical canal. Stimulation by vibration at 0.75 W power and 20,000 times per min vibration was carried out for 30 s per stimulation 7 times at 5 min intervals. The pseudopregnant females produced by mating with vasectomised males were used as controls.

### Analysis of oestrus cycle after artificial stimulation

The oestrus cycles of females after artificial stimulation were observed for 16 days. Vaginal secretions were collected from 10 females and smeared on glass slides. The slides were then stained with 10% Giemsa stain solution. The four stages of the oestrus cycle, proestrus (P), oestrus (E), metestrus (M), and dioestrus (D) were identified. The females without artificial stimulation were used as controls.

### Embryo transfer

The 2-cell embryos were transferred into the oviducts of pseudopregnant females 18–20 h after mating with vasectomised males or artificial stimulation. The pronuclear embryos were transferred into the oviducts of pseudopregnant females 24 h after artificial stimulation. The implantation site was observed and offspring counted at 21 days of gestation.

### Data analysis

Data was analysed using chi-square test followed by a multiple comparison test using Ryan’s method.
